# Calcium responses to external mechanical stimuli in the multicellular stage of *Dictyostelium discoideum*

**DOI:** 10.1038/s41598-022-16774-3

**Published:** 2022-07-20

**Authors:** Hidenori Hashimura, Yusuke V. Morimoto, Yusei Hirayama, Masahiro Ueda

**Affiliations:** 1 Department of Biological Sciences, Graduate School of Science, Osaka University, 1-3 Yamadaoka, Suita, Osaka 565-0871 Japan; 2 RIKEN Center for Biosystems Dynamics Research (BDR), 6-2-3 Furuedai, Suita, Osaka 565-0874 Japan; 3 Graduate School of Arts and Sciences, University of Tokyo, 3-8-1 Komaba, Meguro, Tokyo 153-8902 Japan; 4 Faculty of Computer Science and Systems Engineering, Kyushu Institute of Technology, 680-4 Kawazu, Iizuka, Fukuoka 820-8502 Japan; 5 Japan Science and Technology Agency, PRESTO, 4-1-8 Honcho, Kawaguchi, Saitama 332-0012 Japan; 6 Graduate School of Frontier Biosciences, Osaka University, 1-3 Yamadaoka, Suita, Osaka 565-0871 Japan

**Keywords:** Calcium signalling, Collective cell migration

## Abstract

Calcium acts as a second messenger to regulate many cellular functions, including cell motility. In *Dictyostelium discoideum*, the cytosolic calcium level oscillates synchronously, and calcium waves propagate through the cell population during the early stages of development, including aggregation. In the unicellular phase, the calcium response through Piezo channels also functions in mechanosensing. However, calcium dynamics during multicellular morphogenesis are still unclear. Here, live imaging of cytosolic calcium revealed that calcium wave propagation, depending on cAMP relay, disappeared at the onset of multicellular body (slug) formation. Later, other forms of occasional calcium bursts and their propagation were observed in both anterior and posterior regions of migrating slugs. This calcium signaling also occurred in response to mechanical stimuli. Two pathways—calcium release from the endoplasmic reticulum via IP3 receptor and calcium influx from outside the cell—were involved in calcium signals induced by mechanical stimuli. These data suggest that calcium signaling is involved in mechanosensing in both the unicellular and multicellular phases of *Dictyostelium* development using different molecular mechanisms.

## Introduction

Ca^2+^ signals are essential for many biological activities^1,2^. In multicellular organisms, synchronized elevations of intracellular Ca^2+^ levels ([Ca^2+^]_i_), or bursts, occur in cell populations, which then propagate as waves among cells^3,4^. This phenomenon has been reported in multiple cell types and biological activities, including fertilization in eggs and endothelial wound repair^5,6^. Both [Ca^2+^]_i_ bursts and wave propagation play key roles in orchestrating the activities of multiple cells in vivo and in vitro^2^. Cell–cell communication via Ca^2+^ signaling has been well investigated in animals in which Ca^2+^ waves are propagated by gap-junction communication or paracrine signaling^4^. A common factor evoking [Ca^2+^]_i_ bursts is a mechanical stimulus; transduction of these stimuli into Ca^2+^ signals is mediated by various Ca^2+^ channels including inositol trisphosphate (IP3) receptors, transient receptor potential (TRP) channels, and the stretch-activated ion channel, Piezo^7–12^. These channels are broadly conserved in eukaryotes, including animals, plants, and amoebae^8,11,12^. In mammalian cells, the Piezo channel family members, Piezo1 and Piezo2, regulate mechanosensing, in which diverse cations, including Ca^2+^, flow in response to mechanical stimuli, and are responsible for various biological functions, including touch sensation and cell development^8,9,11^. The genome of the social amoeba, *Dictyostelium discoideum*, encodes a single Piezo homolog, *pzoA*^13^*.* Hence, there is a possibility that Ca^2+^ signaling is universally employed for mechanosensing.

One example of Ca^2+^ wave propagation in populations of eukaryotic cells is cell–cell communication in the aggregation of *Dictyostelium discoideum*. Following starvation, *Dictyostelium* cells aggregate and form cell masses called mounds. Cells in mounds differentiate into either prestalk or prespore cells and form migrating multicellular bodies, or slugs. During aggregation, cAMP signaling, known as cAMP relay, organizes the directed migration of cells^14–16^, and Ca^2+^ waves are propagated simultaneously among starved cells^17^. It has been assumed that cAMP relay is essential for the coordination of collective cell migration throughout *Dictyostelium* development^18,19^; however, recent studies have shown that the dynamics of cAMP signaling are altered after multicellularity begins^16,20^. In addition to Ca^2+^ wave propagation during aggregation^17^, transient [Ca^2+^]_i_ elevation has been observed in mounds and slugs^21^. These results suggest that synchronous [Ca^2+^]_i_ bursts and wave propagation occur not only during aggregation but also in later *Dictyostelium* development, including in the formation of mounds and slugs. Calcium signaling has been suggested to be involved in chemotaxis and stalk cell differentiation^22–26^. However, the dynamics of, and molecular mechanisms underlying [Ca^2+^]_i_ signaling during the morphogenesis of *Dictyostelium* remain unclear.

In this study, [Ca^2+^]_i_ signaling during *Dictyostelium* development was investigated using fluorescent Ca^2+^ probes. This approach revealed the transition of [Ca^2+^]_I_ signaling dynamics during cell aggregation and the formation of slugs of *Dictyostelium*, both of which have robust calcium signaling mechanisms in response to mechanical stimuli.

## Results

### Calcium signaling dynamics change during *Dictyostelium* development

**Figure 1:**
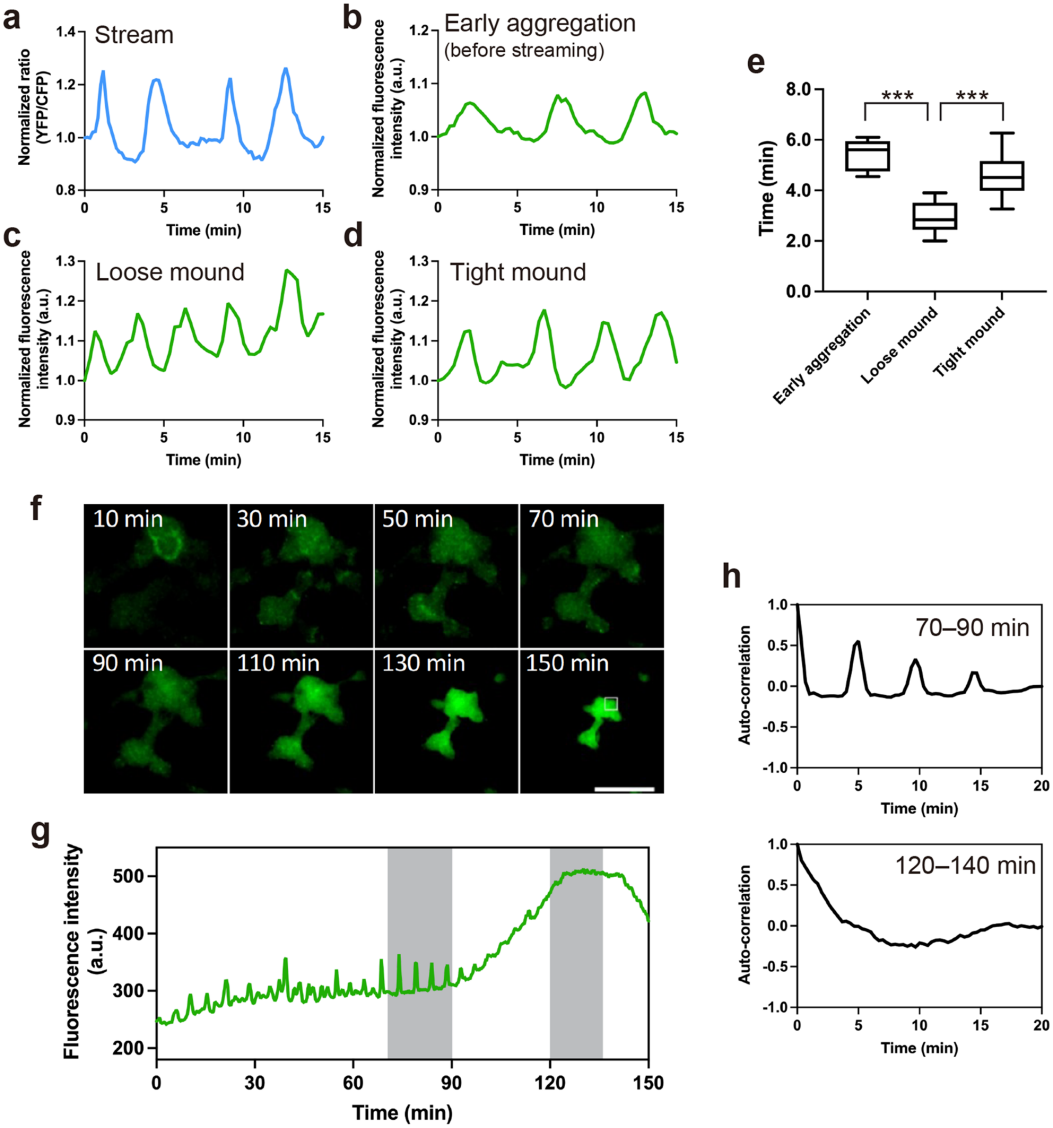
[Ca^2+^]_i_ signal dynamics during development of *Dictyostelium*. Time courses of (**a**) Förster resonance energy transfer (FRET) signals of YC-Nano15 or (**b**–**d**) mean fluorescence intensity of GCaMP6s in a region indicated with a white box (ROI) in Supplementary Fig. 1. Size of ROI: (**a**) 25 μm; (**b**) 250 μm; (**c**) and (**d**) 100 μm. (**a**) Aggregating stream. (**b**) Early aggregation. (**c**) Early (loose) mound. (**d**) Late (tight) mound. (**e**) Boxplot of periods of [Ca^2+^]_i_ oscillations at three developmental stages. The lower and upper error bars indicate smallest and largest values, respectively. For each dataset, n = 13. ***; *p* < 0.001 (ANOVA with Dunnett's T3 multiple comparisons test). (**f**) Fluorescence micrographs of *Dictyostelium* cells expressing GCaMP6s during morphogenesis from the late mound to the finger stage. Scale bar, 500 µm. (**g**) Time course of mean fluorescence intensity of GCaMP6s in a 100 µm^2^ region indicated with the white box in (**f**). (**h**) Autocorrelation of GCaMP6s signals at development stages shown as gray bars in (**g**). Note that the elevation of fluorescence intensity in the entire mound (90–150 min in **f** and **g**) is an artifact of the increase in thickness of the tissue rather than [Ca^2+^]_i_ elevation.

To investigate the relationship between the dynamics of calcium signals and multicellularity in *Dictyostelium,* we monitored [Ca^2+^]_i_ dynamics during development with genetically encoded calcium indicators (GECI). As previously reported^17^, cells expressing the Förster resonance energy transfer (FRET) sensor YC-Nano15 (K_d_ = 15 nM) showed clear oscillations and wave propagation in aggregation streams (Fig. 1a, Supplementary Fig. 1a, and Movie 1). Moreover, [Ca^2+^]_i_ dynamics were investigated with the single-wavelength GECI GCaMP6s^27,28^ to confirm whether the wave propagation observed using YC-Nano15 authentically reflected [Ca^2+^]_i_ dynamics during development using a different GECI, as well as when avoiding the phototoxicity caused by exposure to violet-blue light excitation for YC-Nano15.

In starved *Dictyostelium* cells, [Ca^2+^]_i_ transiently increases in response to external cAMP^22^ and the calcium channel, IplA*,* the homolog of the IP3 receptor, is essential for its elevation^29^. When chemotactic-competent cells expressing GCaMP6s were stimulated by cAMP, wild-type cells showed transient rapid elevations of fluorescence with a peak, 16 s after stimulation; however, cells lacking *iplA* showed no such elevations after stimuli (Supplementary Fig. 2a). Thus, GCaMP6s (K_d_ = 144 nM)^27^ is functional in *Dictyostelium* cells and is appropriate for visualizing [Ca^2+^]_i_ dynamics. Oscillations of fluorescence signals and wave propagation were observed during both early aggregation (before streaming) and mound stages of cells expressing GCaMP6s (Fig. 1b–d, Supplementary Fig. 1b–d, Movies 2–6). These signal oscillations were not observed in *iplA*^−^ cells during development (Supplementary Fig. 2b, c, Movie 7), demonstrating that the periodic changes in GCaMP6s signals in developing *Dictyostelium* cells reflect [Ca^2+^]_i_ oscillations caused by cAMP signal relay. Oscillation periods in the early mound stage were significantly shorter than those in the early aggregation and late mound stages (*p* < 0.001) (Fig. 1e). The early and late mound stages described in this study correspond to the loose and tight mound stages, respectively. The periods of [Ca^2+^]_i_ oscillations in the early aggregation, and early and late mound stages were 5.29 ± 0.59, 2.95 ± 0.61, and 4.60 ± 0.89 min, respectively. These periods are consistent with those of [cAMP]_i_ oscillations^16^. Wave propagation was observed until the late mound stage; however, signal oscillations and propagation in cell populations disappeared when the late mound began elongation, that is, at the onset of slug formation (Fig. 1f–h, Movie 8). Consistent with this, periodic oscillations in the cAMP signal have also been shown to disappear after the late mound stage^16^. Signal visualization using GECIs revealed that [Ca^2+^]_i_ signal dynamics, as well as cAMP signal dynamics, show transitions during multicellular morphogenesis^16^.

### Transient [Ca^2+^]_i_ bursts and their propagation in migrating slugs

**Figure 2:**
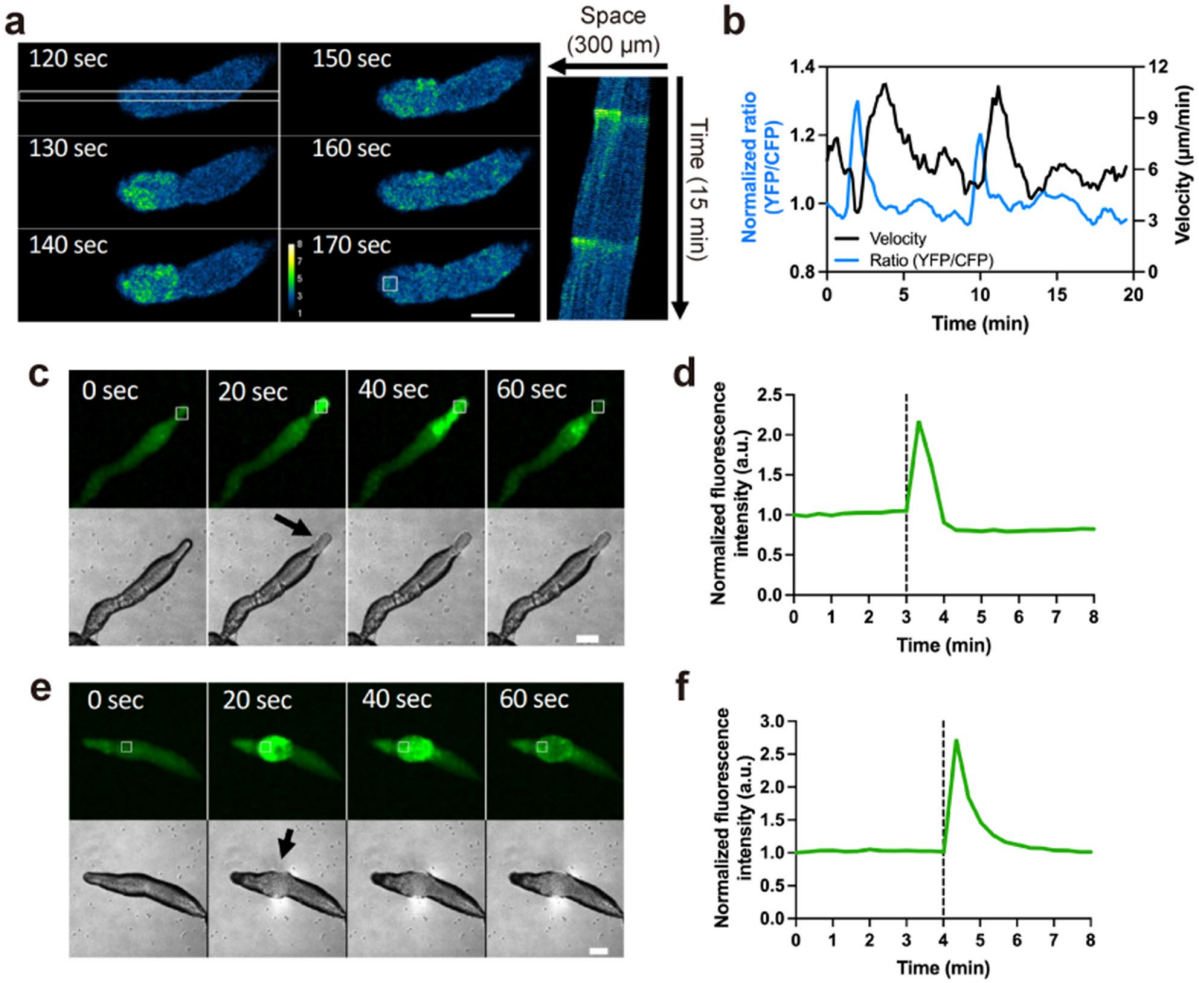
Intracellular Ca^2+^ levels ([Ca^2+^]_i_) burst in *Dictyostelium* slugs during migration. (**a**) Ratiometric images (YFP/CFP) of a slug expressing YC-Nano15 (Left). [Ca^2+^]_i_ burst at a tip of the slug expressing YC-Nano15 and its propagation toward the posterior region of the slug are shown. Scale bar, 50 µm. (Right) Kymograph of [Ca^2+^]_i_ wave propagation in the region containing several cells, indicated with the white rectangle (10 × 300 μm) in the left panel, over 15 min. (**b**) Time course of Förster resonance energy transfer (FRET) signals in the tip of the slug (blue line) and slug velocity (black line). FRET signals of YC-Nano15 in a 15 μm^2^ region in the slug (white box in **a**) was measured. The curves of FRET signals and slug velocity were smoothed using a running average over four data points. (**c**, **e**) [Ca^2+^]_i_ burst at a tip (**c**) or posterior region (**e**) of the slug expressing GCaMP6s. Fluorescence micrographs of GCaMP6s (upper panels) and differential interference contrast (DIC) micrographs (lower panels) are shown. Scale bar, 100 µm. An arrow shows that the slugs are in contact with the agar surface. (**d**, **f**) Time course plot of the mean fluorescence intensity of GCaMP6s in a 50 µm^2^ region indicated with a white box in (**c**) and (**e**). Black dashed lines indicate the time at which the slug was in contact with the agar surface.

During late *Dictyostelium* development, the late mound elongates vertically into a cylindrical structure called a finger, which subsequently falls over and starts to migrate as a slug. When monitoring [Ca^2+^]_i_ dynamics in migrating slugs using YC-Nano15, transient and rapid elevations of [Ca^2+^]_i_, or bursts, and their propagation were observed (Fig. 2a, b, Movie 9), although no wave propagation was observed in the finger stage (Fig. 1). Monitoring the signal using GCaMP6s also revealed these transient signal propagations in migrating slugs, with [Ca^2+^]_i_ bursts observed in both the anterior and posterior of slugs, which are regarded as prestalk and prespore regions, respectively, despite the differences in expressed genes due to differentiation (Fig. 2c–f, Movies 10, 11). During slug [Ca^2+^]_i_ bursts, the migration velocities increased transiently, with a peak delay of approximately 2 min (Fig. 2b, Movie 9). The periodicity of [Ca^2+^]_i_ signals that we observed during aggregation and mound stages (Fig. 1) was not observed in migrating slugs, which occasionally showed irregular [Ca^2+^]_i_ bursts (Fig. 2 and Supplementary Fig. 3). Thus, although periodic [Ca^2+^]_i_ signal propagations disappeared during the process of multicellular development, the ability of Ca^2+^ signaling was maintained after slug formation, and the occasional propagation of [Ca^2+^]_i_ bursts in migrating slugs affected the cooperative movement of cells (Movie 9).

### Slug [Ca^2+^]_i_ bursts are induced by mechanical stimulation

**Figure 3:**
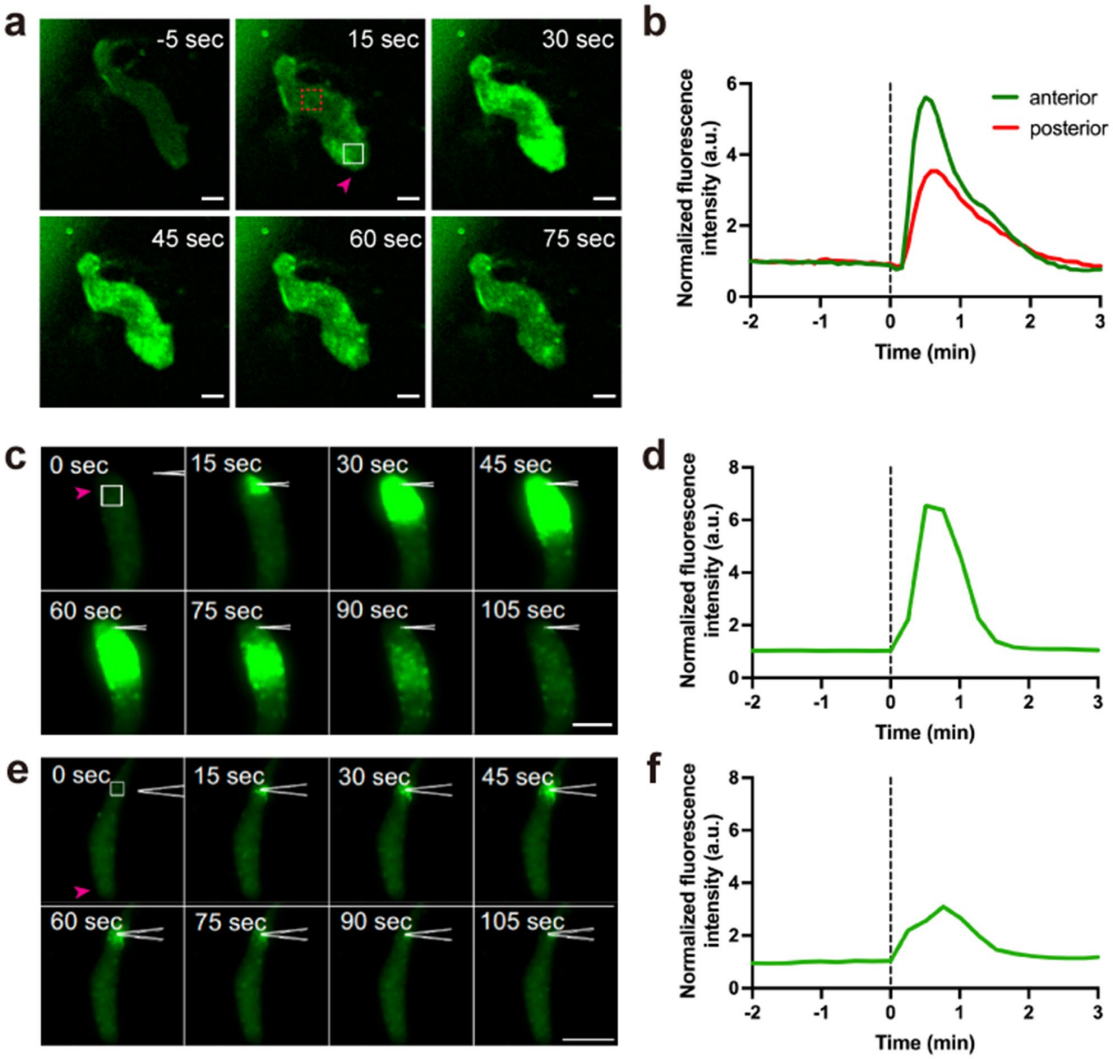
Intracellular Ca^2+^ ([Ca^2+^]_i_) bursts in *Dictyostelium* slugs induced by mechanical stimulation. (**a**) Representative fluorescence micrographs of a slug expressing GCaMP6s, mechanically stimulated with a plastic rod. Scale bar, 50 µm. (**b**) Time course of mean fluorescence intensity of GCaMP6s in ROIs in the anterior (white square) and posterior (red dashed square) regions in (**a**). (**c**, **e**) [Ca^2+^]_i_ bursts at the tip (**c**) or posterior (**e**) of a slug expressing GCaMP6s after mechanical stimulation by pricking with a micropipette. Fluorescence micrographs of slugs expressing GCaMP6s are shown. Anterior part of the slug faces the top (**c**) or bottom (**e**). Scale bar, (**c**), 50 and (**e**), 100 µm. (**d**, **f**) Time course of mean fluorescence intensity of GCaMP6s in the 25 µm^2^ region indicated with the white boxes in (**c**) and (**e**), respectively. Magenta arrowheads in (**a**), (**c**), and (**e**) indicate the anteriors of slugs. Black dashed lines in (**b**), (**d**), and (**f**) indicate the time point of mechanical stimulation.

On closer observation, [Ca^2+^]_i_ bursts in slugs occurred when a part of the slug touched the surface of the agar (Fig. 2c, e and Movies 10, 11). This suggested that rapid [Ca^2+^]_i_ elevation in slugs was induced by mechanical stimuli. To confirm this, [Ca^2+^]_i_ dynamics were monitored using GCaMP6s after slugs were subjected to mechanical stimulation. Slugs developing on agar were excised with their supporting agar, then turned over onto glass dishes, such that they were sandwiched between glass and the agar. They were then pressed from above with a 5 mm diameter plastic rod without crushing so that the entire slug was stimulated evenly (Supplementary Fig. 4a). In all tests using wild-type expressing GCaMP6s, [Ca^2+^]_i_ in the anterior region of slug increased transiently with a peak at 25.0 ± 4.1 s after all stimulation (n = 9) (Fig. 3a, b and Movie 12). The posterior region also responded to the stimulus with the same peak time as the anterior region, but with attenuated changes in [Ca^2+^]_i_ levels (Fig. 3b). In contrast, neither an increase nor a decrease in intracellular cAMP concentration was observed when slugs expressing the cAMP fluorescent probe, Flamindo2, were stimulated (Supplementary Fig. 5). Additionally, when slug tips were pricked using a micropipette (Supplementary Fig. 4b), [Ca^2+^]_i_ bursts were induced and signals were propagated posteriorly (Fig. 3c, d and Movie 13). A similar response was observed, but with lower changes in [Ca^2+^]_i_ levels, when the posterior region of the slug was stimulated (Fig. 3e, f and Movie 14). These indicate that [Ca^2+^]_i_ burst in the slug occurs in response to mechanical stimulation applied to either the anterior or posterior of the slug.

### The IplA Ca^2+^ channel is involved in calcium signaling in response to the mechanical stimulation of slugs

**Figure 4:**
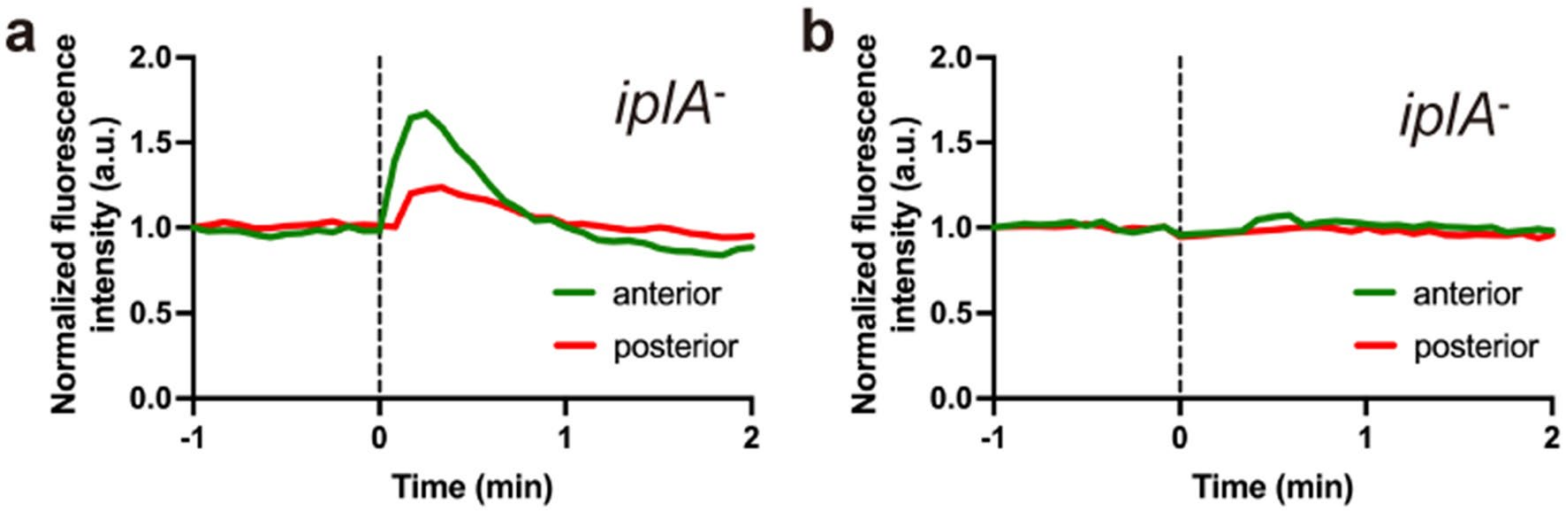
Intracellular Ca^2+^ ([Ca^2+^]_i_) bursts in *iplA*^−^ slugs induced by mechanical stimulation. [Ca^2+^]_i_ was monitored in *iplA*^−^ slugs expressing GCaMP6s after mechanical stimulation with a plastic rod. (**a**) Representative time courses of the mean fluorescence intensity of GCaMP6s in the anterior (green) and posterior (red) regions of a slug showing [Ca^2+^]_i_ bursts. (**b**) Representative time courses of mean fluorescence intensity of GCaMP6s in a slug showing no [Ca^2+^]_i_ response. Black dashed lines indicate the time of mechanical stimulation.

In the unicellular phase of *Dictyostelium*, the IP3 receptor, IplA, which is localized in the endoplasmic reticulum (ER), is responsible for [Ca^2+^]_i_ elevation in response to mechanical stimuli^30^. Expression of *iplA* mRNA is low during growth, peaks at about 9 h after starvation, and then decreases^29^. To confirm whether IplA is involved in [Ca^2+^]_i_ bursts induced by mechanical stimuli in slugs, we measured [Ca^2+^]_i_ responses to mechanical stimuli in slugs lacking *iplA*. When *iplA*^−^ slugs were stimulated with a plastic rod, [Ca^2+^]_i_ bursts occurred; however, even within the same slug, the incidence of responses dropped to 46% (18/39 trials in 16 slugs), and the response at the anterior region peaked at 15.7 ± 7.9 s (n = 18), earlier than in wild-type controls (Figs. 3 and 4). There was no difference in the timing of the response peak between the anterior and posterior regions (Fig. 4). Calcium responses were also observed when wild-type and *iplA*^−^ slugs bumped into the agar (Supplementary Fig. 6). These results suggest that [Ca^2+^]_i_ bursts in response to mechanical stimuli are partially mediated by the IplA channel.

### Calcium influx allows for a rapid response to mechanical stimuli

**Figure 5:**
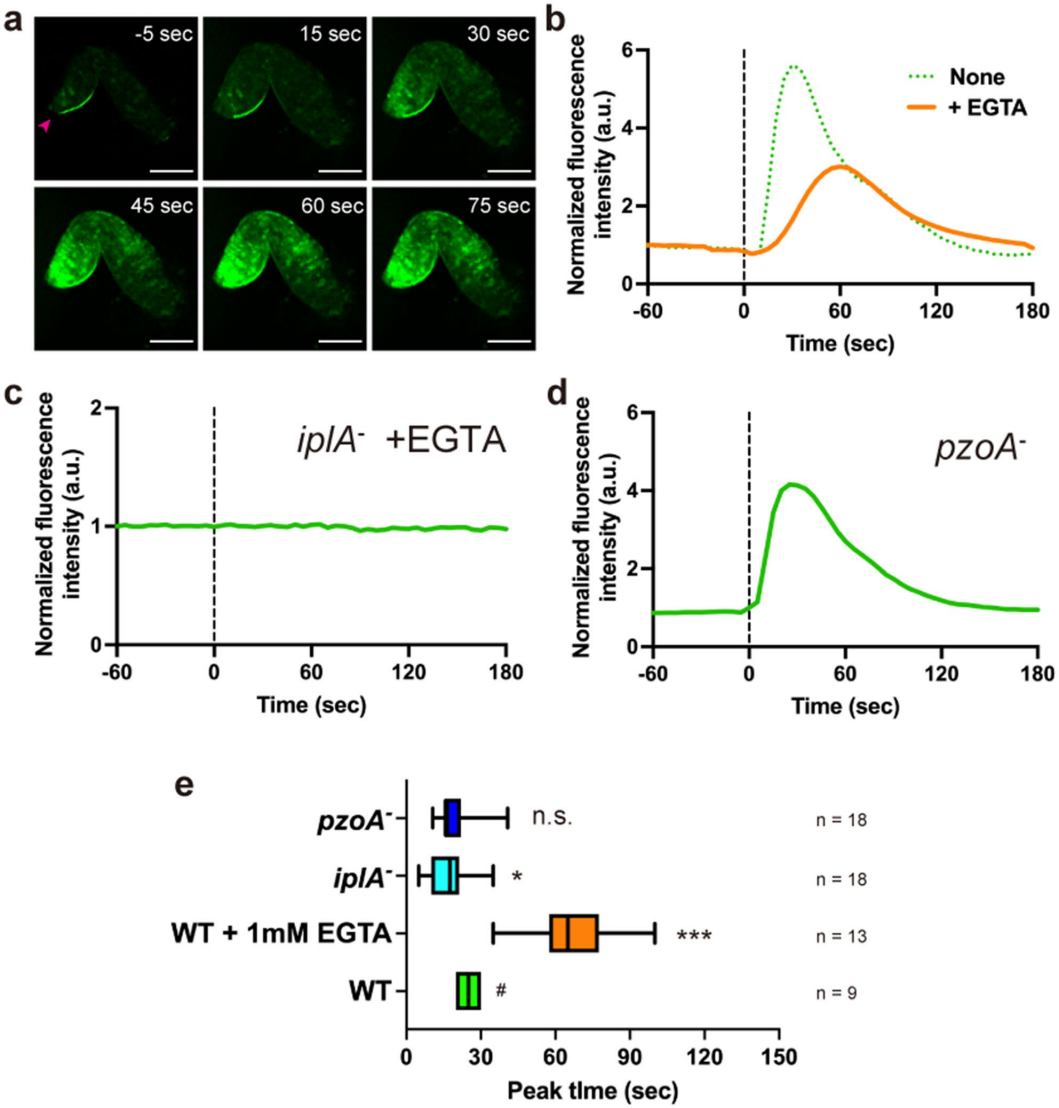
Effect of extracellular Ca^2+^ on intracellular Ca^2+^ ([Ca^2+^]_i_) bursts in *Dictyostelium* slugs induced by mechanical stimulation. (**a**) Wild-type slug covered with a piece of agar containing 1 mM aminoethyl ether)-*N*,*N*,*N*′,*N*′-tetraacetic acid (EGTA). Representative fluorescence of GCaMP6s in an *iplA*^−^ slug, mechanically stimulated with a rod on EGTA agar. Magenta arrowhead indicates the anterior of the slug. Scale bar, 100 µm. (**b**) Orange line shows time course of mean fluorescence intensity of GCaMP6s in the 25 µm^2^ ROI in the anterior region in A. The green dashed line shows fluorescence intensity in the anterior region in a slug without EGTA in Fig. 3b. (**c**) Time course of mean fluorescence intensity of GCaMP6s in *iplA*^−^ slugs with agar containing 1 mM EGTA. (**d**) Time course of mean fluorescence intensity of GCaMP6s in PzoA null slugs without 1 mM EGTA. (**e**) Box plots of the peak [Ca^2+^]_i_ bursts elicited by mechanical stimuli. Lower and upper error bars indicate smallest and largest values, respectively. Average values were compared with the peak time of the wild-type slug without EGTA (#). *, *p* < 0.05; ***; *p* < 0.001 (ANOVA with Dunnett's T3 multiple comparisons test). *n.s.* indicates no significant difference.

Deletion of IplA did not completely abolish the slug calcium response to mechanical stimulation (Fig. 4), indicating that another Ca^2+^ pathway contributes to mechanosensing. It has been reported that extracellular Ca^2+^ influx via the Piezo channel homolog is important for mechanosensing during the unicellular stage of *Dictyostelium*^31^. To investigate whether Ca^2+^ influx occurs during multicellular stages, calcium response was monitored on agar medium containing ethylene glycol-bis(β-aminoethyl ether)-N,N,N′,N′-tetraacetic acid (EGTA) to chelate Ca^2+^. All slugs overlayed with agar containing 1 mM EGTA showed an increase in [Ca^2+^]_i_ in response to mechanical stimuli (n = 13) (Fig. 5a, b). However, EGTA slowed the response peak to 67.7 ± 16.8 s (*p* < 0.001) (Fig. 5b, e). Additionally, in the presence of 1 mM EGTA, *iplA*^−^ slugs did not show any calcium response to mechanical stimulation (n = 13) (Fig. 5c). These and our *iplA*^−^ mutant results indicate that Ca^2+^ influx from extracellular sources allows a fast response, whereas IplA is essential for the response from intracellular sources (Figs. 4 and 5c). We constructed a *pzoA* null strain and confirmed that PzoA is essential for Ca^2+^ influx from extracellular sources by mechanical stimulation during unicellular stages as previously reported (Supplementary Fig. 7)^31^. However, the response of *pzoA*^−^ slugs was similar to that of wild-type, suggesting that Ca^2+^ influx is mediated by other pathways during the multicellular phase (Fig. 5d, e).

## Discussion

In *Dictyostelium*, [Ca^2+^]_i_ transients have been observed during both the mound and slug stages^21^; however, the actual dynamics of Ca^2+^ signaling have not been clarified, because most previous studies have focused on stages before aggregation^17,23,29,32^. In this study, [Ca^2+^]_i_ imaging during *Dictyostelium* development with highly sensitive GECIs revealed that synchronized [Ca^2+^]_i_ elevations and their propagation in cell populations occur continuously during the aggregation and mound stages; however, they disappear in the late stage of multicellular development. Ca^2+^ wave propagation depends on cAMP relay during the early aggregation and mound stages, and cAMP wave propagation disappears during tip elongation in the late mound^16,20^. This cAMP signal has been shown to induce transient [Ca^2+^]_i_ elevation^23,33^. Therefore, changes in Ca^2+^ dynamics of during development follow the transition of cAMP relay. We found that [Ca^2+^]_i_ bursts and their propagation occasionally occurred in slugs (Fig. 2). Consistent with previous reports of cAMP signaling^16^, the signal oscillation periods in the early mound stage was shorter than in the early aggregation and late mound stages (Fig. 1e). The decrease in the oscillation periods is due to increased cell density and extracellular cAMP levels^34,35^, and the increase in the oscillation periods can be explained by the expression of low-affinity cAMP receptors in the mound stage instead of the high-affinity cAMP receptors expressed in the aggregation stage^36^. Our results suggest that these occasional [Ca^2+^]_i_ bursts are in response to mechanical stimulus caused by bumping of the tips of migrating slugs in an environment where they are sandwiched between agar and glass (Supplementary Fig. 4). When [Ca^2+^]_i_ signals were propagated in migrating slugs, migration velocity transiently increased. Ca^2+^ signaling affects both cell movement at the unicellular stage and slug behavior^30,37,38^. Calcium wave propagation and its effects are also well known in animal cells, with gap junctions as essential components in cell–cell signaling^4^. Given that *Dictyostelium* does not have gap junction component homologs^39,40^, the mechanism for calcium signal propagation in slugs must be gap junction independent.

*Dictyostelium* cells show [Ca^2+^]_i_ elevation in response to cAMP signals and mechanical stimuli in the unicellular phase^30,31,41^. Our assay showed that slug [Ca^2+^]_i_ bursts and their propagation were induced by mechanical stimuli. The IplA Ca^2+^ channel and Ca^2+^ release from ER have been shown to be involved in the Ca^2+^ response to mechanical stimuli in the unicellular phase^30,31,41,42^. In an *iplA*^−^ strain, there is no elevation of [Ca^2+^]_i_ in response to cAMP stimulation of starved single cells and cell aggregation is delayed^29,43^. In slugs lacking IpIA, the calcium response to mechanical stimulation was attenuated, suggesting that IplA is responsible for increasing the certainty of response to mechanical stimuli. The IplA channel has been shown to be essential for Ca^2+^ dependent flow-directed motility, but not for chemotactic migration toward cAMP sources^44^. This suggests that both cAMP signaling and IplA-mediated Ca^2+^ signaling affect downstream components independently. This hypothesis is supported by the observation that there is no clear defect in the development of *iplA*^−^ slugs under laboratory conditions (Movie 7). However, mechanosensing may play important morphogenetic roles in natural environments, where soil-living *Dictyostelium* amoebae are exposed to more complex stimuli and physical barriers^45^. Translocation of a transcription factor has been demonstrated during the repair of slugs in response to mechanical damage^36^. Therefore, a Ca^2+^ response to mechanical stimulation requiring IplA might only be important in natural environments (Movie 2). Alternatively, the Ca^2+^ response to mechanical stimulation was not completely abolished in *iplA*^*−*^ slugs, suggesting that other pathways are involved in the elevation of [Ca^2+^]_i_. In the *Dictyostelium* genome, other potential Ca^2+^ signaling components include the mucolipin channel (*mcln*), two pore channels (*tpc*), a transient receptor potential (*trp*) channel, and an Msc-like channel (*mscS*)^42,46–49^. In higher eukaryotes, the stretch-activated Ca^2+^ permeable ion channel Piezo is involved in mechanical stimulus responses^8,9^. Recently, it has been reported that *D*. *discoideum* has a homolog of Piezo, PzoA. Disruption of the *pzoA* gene causes defects in the [Ca^2+^]_i_ response to mechanical stimuli in amoebae^31^. Additionally, cells lacking PzoA develop normally under laboratory conditions; however, a defect is present in chemotactic migration under confined conditions^31^. Slugs lacking *pzoA* did not show a substantial difference in calcium response from that of wild-type control slugs. Notably, the Piezo channel in higher multicellular organisms functions only in the unicellular phase in *Dictyostelium*. Even within *Dictyostelium*, there are interesting changes, with Piezo acting as the main pathway during unicellularity, and pathways from the extracellular and ER during multicellularity. In *iplA*^−^ cells, the [Ca^2+^]_i_ response was faster than in the wild type. This indicates that the apparent single-peak [Ca^2+^]_i_ burst is a mixture of a fast extracellular Ca^2+^ influx with a slower, yet more efficient, response from the ER. This delayed ER response may be due to the fact that the signal from mechanoreceptors in the plasma membrane is transmitted via IP3. Moreover, in the unicellular phase of *iplA*^−^ cells, no calcium response to mechanical stimuli was detected, even though IplA is not involved in blebbing, which is regulated by calcium signaling related to mechanical stimulation^31^. In a human colorectal carcinoma cell line, membrane blebbing is regulated by store-operated Ca^2+^ entry, which is controlled by ER proteins^50^. Alternatively, although no homolog of stromal interaction molecule (STIM) has been found in *Dictyostelium*^51^, unknown store-operated calcium channels (SOCs) may be transducing mechanical stimuli.

The [Ca^2+^]_i_ bursts at both the tip and posterior regions in slugs indicate the ability for rapid [Ca^2+^]_i_ elevation in both prestalk and prespore cells. Previous studies have shown that [Ca^2+^]_i_ in the anterior part of the slug is higher than that in the posterior^21,22^. However, in our study, this difference was not observed in slugs in a steady state, not receiving any external stimuli. On the contrary, if these previous data contain dynamic information, they are consistent with those of our study (Fig. 3). As slugs migrate with their tips protruding vertically^52^, it may be easier to generate anterior responses to obstacles or enemies, such as nematodes^53^. Thus, it has been frequently observed that [Ca^2+^]_i_ is higher in the anterior than in the posterior of the slug. Because Ca^2+^ responses were observed when slugs collapsed (Fig. 2c–f), it is also possible that the mechanical stimulus response supports the construction of the fruiting body.

In conclusion, we have shown that [Ca^2+^]_i_ bursts and their propagation in *Dictyostelium* are dependent on cell–cell communication via diffusible chemical signals during early developmental stages. Following multicellular development, such Ca^2+^ signaling is triggered by mechanical stimuli. Because mechanical stimuli could not be accurately quantified in our system, the contribution of calcium to the mechanical stimulus response remains unresolved and warrants further study. Ca^2+^ signaling in response to mechanical stimuli is conserved broadly in higher eukaryotes and prokaryotes, such as *Escherichia coli*^4,54–56^. We observed that the social amoeba, *D*. *discoideum*, belonging to Amoebozoa, uses Ca^2+^ signaling as a mechanosensing signal in the multicellular phase similar to that in the unicellular phase^31^; however, the molecular mechanism is different. Thus, this study demonstrates that mechanochemical signal transduction via Ca^2+^ signaling is a universal system for response to mechanical stimuli and can be applied in any cell type or state. In this study, the pathway for extracellular Ca^2+^ uptake associated with mechanical stimulation in the slugs of *D*. *discoideum* was not identified. This is because even though Ca^2+^ acts as a signal across species, molecular mechanisms differ significantly. Further studies are required to clarify conserved and specific molecular mechanisms.

## Methods

### Strains and culture conditions

*Dictyostelium discoideum* strains used are listed in Supplementary Table 1. The *pzoA*^*−*^ strain was constructed using the vector pKOSG-IBA-dicty1 (iba) following the manufacturer’s instructions^57^. The 5′- and 3′-flanking sequences were generated using polymerase chain reaction (PCR) and cloned into pKOSG-IBA-dicty1 (Supplementary Fig. 7a). Primer pairs used for PCR were pzA_KO_LA1/pzA_KO_LA2 (5′-) and pzA_KO_RA1/pzA_KO_RA2 (3′-) (Supplementary Table 2). The *pzoA* gene disruption was confirmed via PCR. Cells were grown axenically in HL5 medium (Formedium, UK) in culture dishes at 21 °C. Transformants were maintained in 20 μg mL^−1^ G418 or 10 μg mL^−1^ blasticidinS (both from Fujifilm Wako, Japan).

### Plasmid construction and genetic manipulation

Plasmids are listed in Supplementary Table 3. pHK12neo_Dd-GCaMP6s was constructed by inserting synthesized GCaMP6s fragments (GenScript) into the BglII and SpeI sites of pHK12neo. The codon usage of the GCaMP6s sequence was optimized to that of *D*. *discoideum* for efficient protein expression. The wild-type and mutant cells were transformed with ~ 1.5 μg plasmid via electroporation^58^, and transformants were selected using G418 or blasticidinS.

### Imaging

In all experiments, cells were observed at 22 °C. Confocal images were taken using an A1 confocal laser microscope (Nikon, Japan) with an oil immersion lens (Plan Fluor 40 ×/1.30 NA, Nikon), or using an inverted microscope (Eclipse Ti, Nikon) equipped with a CSU-W1 confocal scanner unit (Yokogawa), two sCMOS cameras (ORCA-Flash4.0v3, Hamamatsu Photonics, Japan), and oil immersion lenses (Plan Apo 60 ×/1.40 NA or CFI Apo TIRF 60 ×/1.49, Nikon). GCaMP6s and YC-Nano15 were excited using 488 and 440 nm solid-state CW lasers, respectively. Epifluorescence micrographs were acquired using an inverted epifluorescence microscope (IX83, Olympus, Japan) equipped with a 130 W mercury lamp system (U-HGLGPS, Olympus), sCMOS cameras (Zyla4.2, Andor Technology or Prime 95B, Photometrics, USA), and objective lenses (UPLSAPO 4 ×/0.16 NA, UPLSAPO 10 ×/0.40 NA, UPLSAPO 20 ×/0.75 NA, Olympus). Cells expressing GCaMP6s or Flamindo2 were observed using fluorescence mirror units U-FGFP (Excitation BP 460–480, Emission BP 495–540, Olympus). All images were processed and analyzed using the Fiji^59^ and R software. GCaMP6s oscillations were calculated by averaging differences between peaks. Data with at least three peaks in the oscillation were used for the analysis. In general, fluorescence intensities of GCaMP6s, Flamindo2, and the ratio of YFP/CFP channels of YCNano15 were normalized to their values at t = 0.

### Live imaging of [Ca^2+^]_i_ dynamics during *Dictyostelium* development

Imaging was performed as described previously^16^. To induce development upon starvation, exponentially growing cells (1.5–3 × 10^6^ cells ml^−1^) were harvested and washed three times in KK2 phosphate buffer (20 mM KH_2_PO_4_/K_2_HPO_4_, pH 6.0). To monitor development, cells were plated on the entire surface of 2% water agar (2% w/v Difco Bacto-agar in ultrapure water) in 35 mm plastic dishes at a density of 5–7 × 10^5^ cells cm^−2^ (Iwaki, Japan) and incubated at 21 °C. Thereafter, plates were filled with liquid paraffin (Nacalai Tesque, Japan) to attenuate light scattering and for microscopy. Additionally, the “2D slug” method^60,61^ was applied for observing slug migration without three-dimensional scroll movement (Fig. 4a and Supplementary Fig. 2). One microliter of cell suspension (4 × 10^7^ cells mL^−1^) was dropped on 2% water agar plates with 2 μL liquid paraffin. A coverslip was placed over the suspension and incubated at 21 °C for a minimum of 15 h.

### GCaMP6s as an indicator of [Ca^2+^]_i_

*Dictyostelium* cells expressing GCaMP6s were suspended in 1 mL developmental buffer (DB: 5 mM Na/KPO_4_, 2 mM MgSO_4_, 0.2 mM CaCl_2_, pH 6.5) at a density of 5 × 10^5^ cells mL^−1^ and incubated for 1 h. Thereafter, cells were exposed to 100 nM cAMP pulses at 6 min intervals for the next 5 h. Following starvation with cAMP pulses, cells were washed three times with 1 mL DB and resuspended in DB at a density of 10^6^ per mL. Cell suspension (40 µL) was deposited onto a glass bottom dish. Cells were stimulated by adding 160 μL of 12.5 μM cAMP (Sigma Aldrich, USA) to the cell droplet (final concentration 10 μM). During stimulation, confocal fluorescent micrographs of starved cells were acquired at 5 s intervals. Averaged fluorescence intensities of GCaMP6s in 5 μm^2^ regions positioned within the cytosol were estimated for each time point.

### Slug [Ca^2+^]_i_ response to mechanical stimulation

Five microliter of cell suspension, at a density of 4 × 10^7^ cells mL^−1^, was deposited on 2% water agar with or without 1 mM EGTA and incubated at 21 °C for 12–15 h. Following slug formation, a piece of agar containing slugs was excised and placed upside down on a spacer attached to a 35 mm glass bottom dish (12 mm diameter glass, Iwaki). The spacer was filled with liquid paraffin to prevent desiccation during observation and to avoid light scattering. Slugs covered with agar were pushed with a 5 mm diameter plastic rod using a micromanipulator system (MM-94 and MMO-4, Narishige, Japan) (Supplementary Fig. 4a). In the micropipette assay, a piece of agar with slugs was excised and placed directly on a 35 mm glass bottom dish (12 mm diameter glass, Iwaki). A wet paper was placed in the dish and the agar piece was covered with liquid paraffin. A Femtotip microcapillary (1 µm tip diameter, Eppendorf, Germany) was mounted onto a Femtojet pump and micromanipulator (Eppendorf). The slug was pricked with the pipette using manual operation with the manipulator (Supplementary Fig. 4b). During mechanical stimulation, images of slugs expressing GCaMP6s were acquired at 5 s intervals using epifluorescence microscopy.

## Supplementary Information

Supplementary Figures.

Supplementary Video 1.

Supplementary Video 2.

Supplementary Video 3.

Supplementary Video 4.

Supplementary Video 5.

Supplementary Video 6.

Supplementary Video 7.

Supplementary Video 8.

Supplementary Video 9.

Supplementary Video 10.

Supplementary Video 11.

Supplementary Video 12.

Supplementary Video 13.

Supplementary Video 14.

Supplementary Video 15.

## Data Availability

The data supporting the findings of this study are available from the corresponding author upon request.
