# Supplementary material for: Calcium responses to external mechanical stimuli in the multicellular stage of Dictyostelium discoideum
Source: Sci Rep. 2022 Jul 20;12:12428. doi: 10.1038/s41598-022-16774-3 (PMC9300675; doi:10.1038/s41598-022-16774-3)
Supplement: Supplementary file 1 — Supplementary Figures. [file 41598_2022_16774_MOESM1_ESM.pdf]

Supplementary Figures

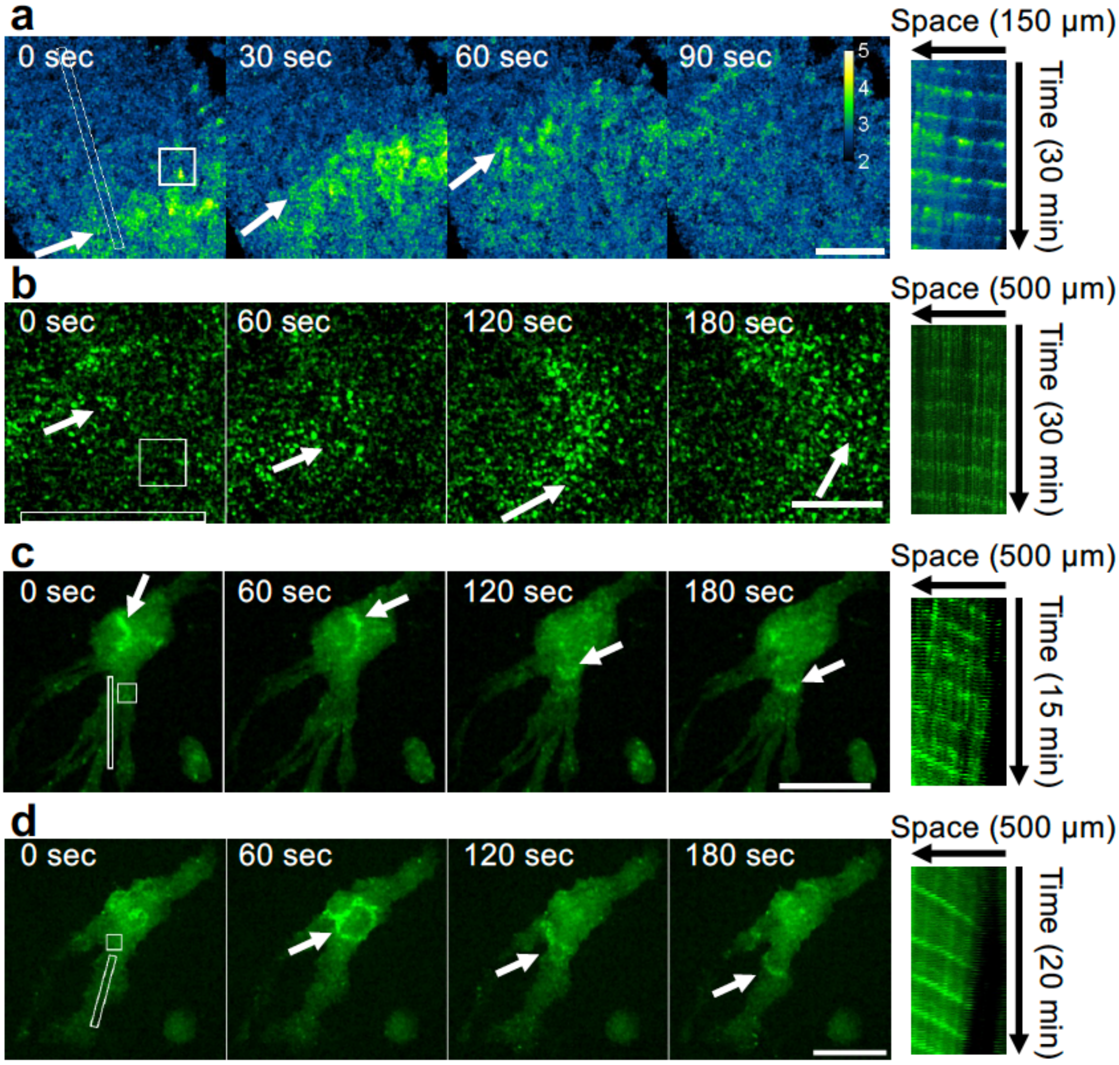

Supplementary Figure 1. Typical intracellular Ca^{2+} levels ($[Ca^{2+}]_i$) wave propagation at each developmental stage of *Dictyostelium* cells. Left panels show $[Ca^{2+}]_i$ wave propagation at each developmental stage. Ratiometric images (YFP/CFP) of cells expressing YC-Nano15 (a) and fluorescence images of cells expressing GCaMP6s (b–d) are shown. White arrows indicate the positions of waves. Right panels show the kymograph of $[Ca^{2+}]_i$ wave propagation in the region indicated by a white rectangle (a and b, 50 × 1000 µm; c and d, 25 × 500 µm) in the left panel. Duration of the kymographs is 30 (a and b), 15 (c) and 20 min (d). (A) An aggregating stream. (b) Early aggregation. (c) An early mound. (d) A late mound. Scale bar; a, 50 µm, b–d, 500 µm.

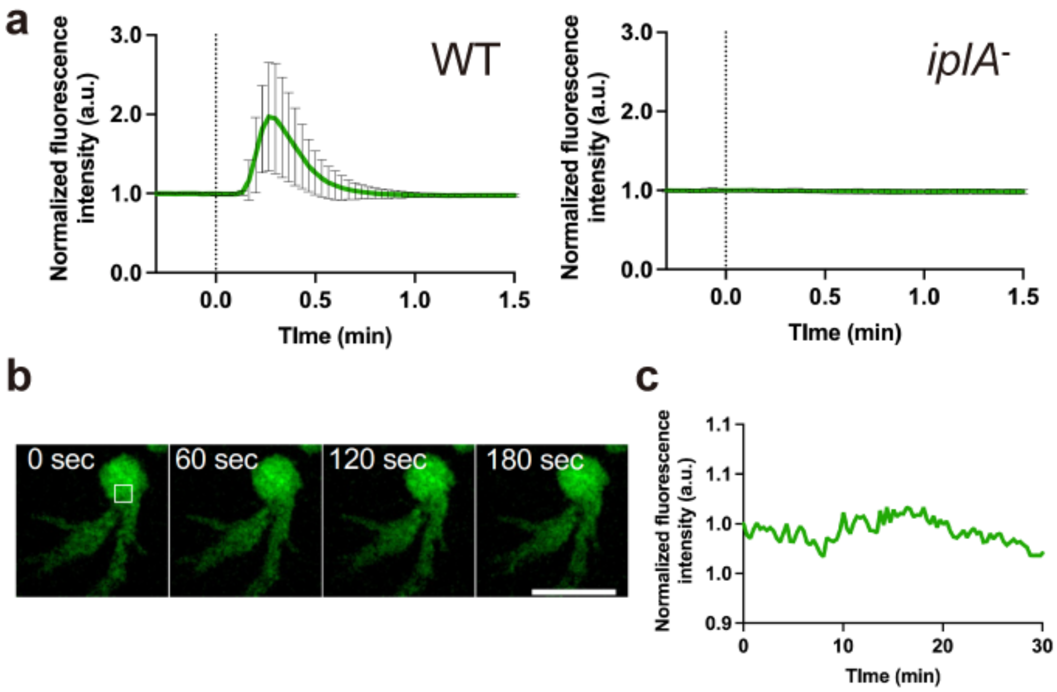

Supplementary Figure 2. Detection of cAMP-dependent intracellular Ca^{2+} level ($[Ca^{2+}]_i$) elevation in starved *Dictyostelium* cells using GCaMP6s. (a) Time-course plots of GCaMP6s signals in *Dictyostelium* cells starved for 5 hours (left, wild type; right, *iplA*$^-$) after 10 µM cAMP stimulation. The mean fluorescence intensity of GCaMP6s in the 5 µm^2 region on cells is plotted on the y-axis (mean ± SD; n = 10 cells in each graphs). Dashed lines indicate the time point of stimulation. (b) $[Ca^{2+}]_i$ signal dynamics during the development of *iplA*$^-$ cells. The fluorescence images of an early mound of *iplA*$^-$ cells expressing GCaMP6s are shown. Scale bar, 500 µm. (c) The time course plot of the mean fluorescence intensity of GCaMP6s in a 100 µm^2 region indicated by a white box in (b).

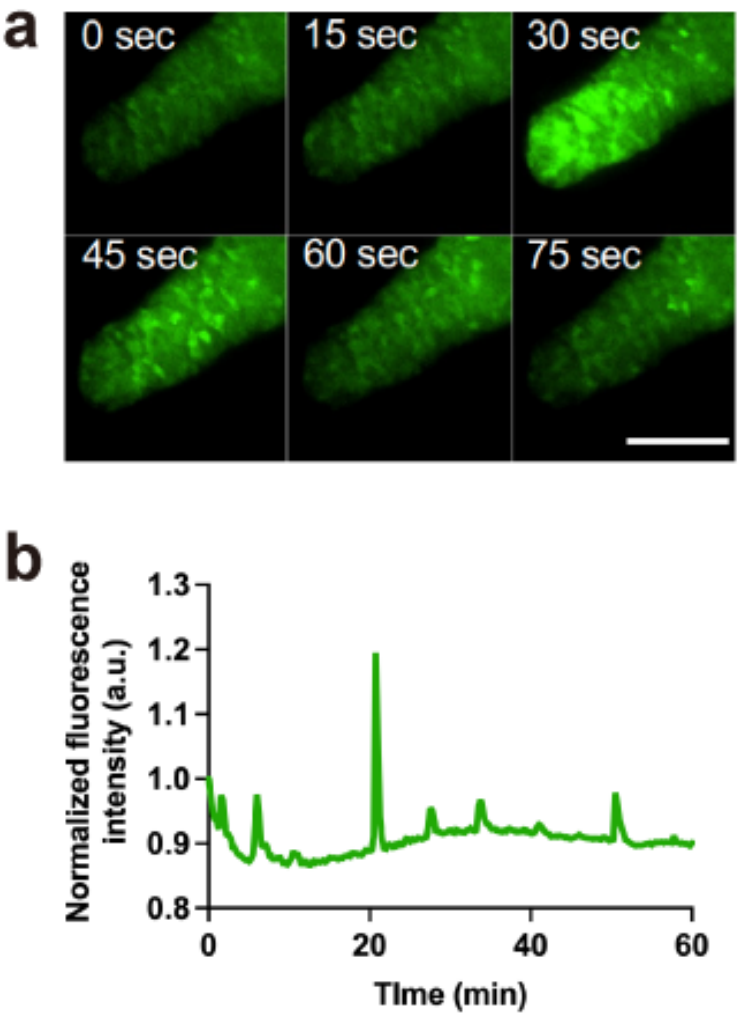

Supplementary Figure 3. Non-periodic intracellular Ca^{2+} levels ($[Ca^{2+}]_i$) burst in *Dictyostelium* slugs. (a) $[Ca^{2+}]_i$ burst at the tip of a wild-type slug expressing GCaMP6s. High magnification fluorescence images of the slug expressing GCaMP6s are shown. Scale bar, 50 μm. (b) The time course plot of mean fluorescence intensity of GCaMP6s in a whole region of the slug in A.

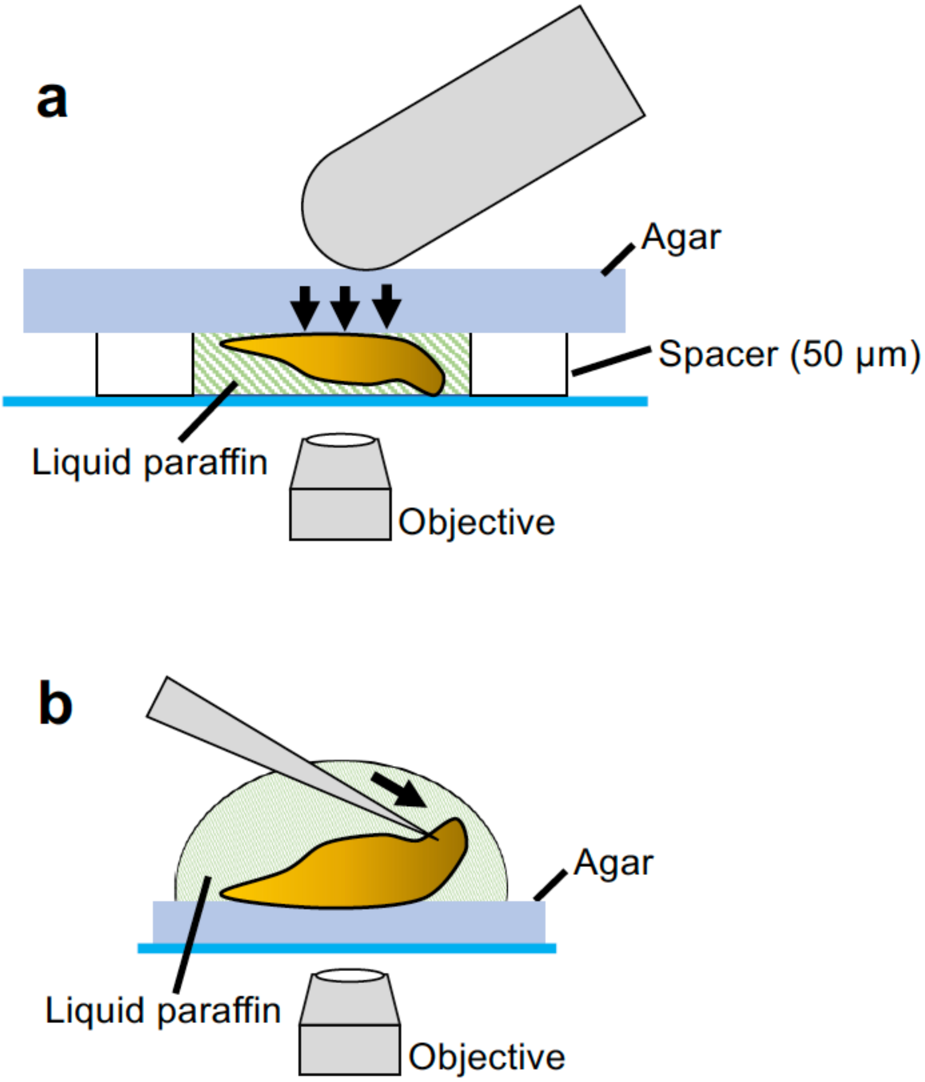

Supplementary Figure 4. Schemes of mechanical stimulation of slugs. (a) The broad stimulation of slugs. A slug was placed between a coverslip and agar sheet and pressed by pushing the agar sheet with a plastic rod. (b) The “sharp” mechanical stimulation. A micropipette was pricked to the slug.

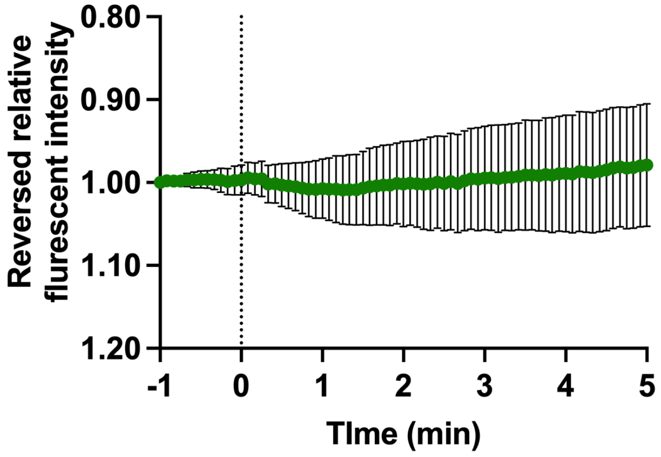

Supplementary Figure 5. Intracellular cAMP signal in slug following mechanical stimulation. Time-course plots of Flamindo2 signals in wild-type slugs after mechanical stimulation with a plastic rod (as in Supplementary Figure 4a). The mean fluorescence intensity of Flamindo2 in the anterior regions on seven slugs is plotted (mean ± SD). Dashed line indicates the time point of the stimulation.

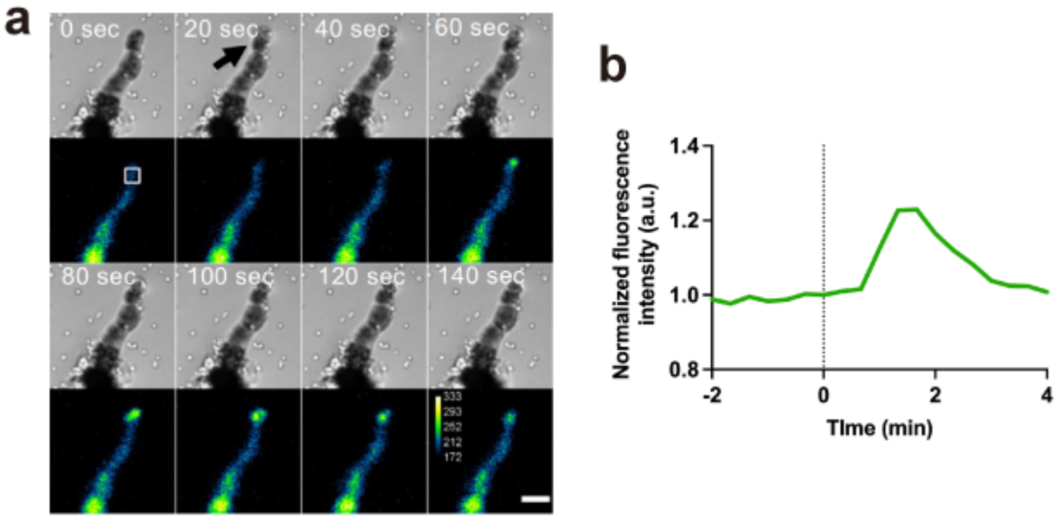

Supplementary Figure 6. Weak intracellular Ca^{2+} levels ($[Ca^{2+}]_i$) burst in *iplA*$^-$ slug induced by mechanical stimulation. (a) $[Ca^{2+}]_i$ burst at a tip of a *iplA*$^-$ slug expressing GCaMP6s. Fluorescence images of GCaMP6s (lower panels) and differential interference contrast (DIC) images (upper panels) are shown. Scale bar, 100 μm. An arrow shows that slugs are in contact with the agar surface. (b) Time course plot of the mean fluorescence intensity of GCaMP6s in a 50 μm^2 region indicated by a white box in (a). A black dashed line indicates the time point when the slug made contact with the agar surface.

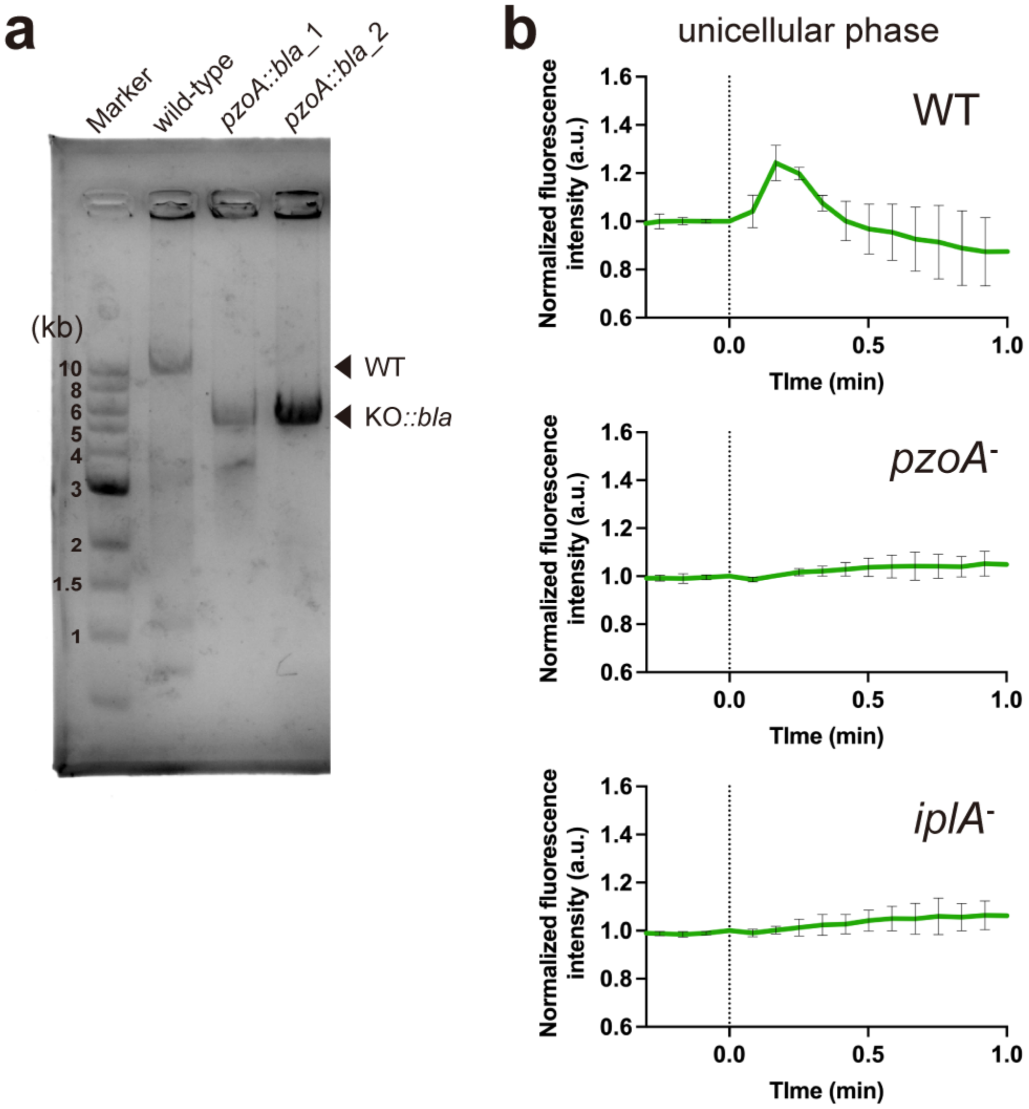

Supplementary Figure 7. Confirmation of PzoA knock out (KO) strain. (a) Piezo channel homolog gene *pzoA* was disrupted by homologous recombination in AX2. A gel electrophoresis of PCR product shows that the *pzoA* gene has been replaced with the blasticidin cassette in two clones of PzoA KO strain. (b) Time-course plots of GCaMP6s signals in starved *Dictyostelium* cells in DB (upper panel, wild-type; middle panel *pzoA*$^-$ cell; lower panel, *iplA*$^-$ cell) after mechanical stimulation with plastic rod (as in Supplementary Fig. 4a). The mean fluorescence intensity of GCaMP6s in the 5 μm^2 region on cells is plotted (mean ± SD). Dashed lines indicate the time point of the stimulation.

Movies

Movie 1
$[Ca^{2+}]_i$ wave propagation in aggregating streams visualized with YC-Nano15 (see Supplementary Fig. 1a for corresponding snapshots). Fluorescent images of YC-Nano15 were acquired by a confocal microscope at 10 s intervals. Ratio of CFP/YFP signals is shown by pseudocolor. Scale bar, 50 μm.

Movie 2
Live imaging of $[Ca^{2+}]_i$ dynamics through the development of *Dictyostelium* cells using GCaMP6s (first half). Data were obtained 2–7 hours after starvation. This movie shows the onset of aggregation, early aggregation and the early mounds with streams. Left, DIC image. Right, fluorescence image of GCaMP6s. The images were acquired by an epifluorescence microscope at 20 s intervals. Scale bar, 1 mm.

Movie 3
Live imaging of $[Ca^{2+}]_i$ dynamics through the development of *Dictyostelium* cells using GCaMP6s (latter half). Data were obtained 7–24 hours after starvation. This is a continuation from Movie 2. This movie shows the late mounds, finger formation, slug migration and fruiting body formation. Left, DIC image. Right, fluorescence image of GCaMP6s. The images were acquired by an epifluorescence microscope at 20 s intervals. Scale bar, 1 mm.

Movie 4
$[Ca^{2+}]_i$ wave propagation at early aggregation visualized with GCaMP6s (see Supplementary Fig. 1b for corresponding snapshots). Fluorescent images of GCaMP6s were acquired by the epifluorescence microscope at 10 s. Scale bar, 50 μm.

Movie 5
$[Ca^{2+}]_i$ wave propagation in an early mound visualized with GCaMP6s (see Supplementary Fig. 1c for corresponding snapshots). Fluorescent images of GCaMP6s were acquired by the epifluorescence microscope at 20 s intervals. Scale bar, 500 μm.

Movie 6
$[Ca^{2+}]_i$ wave propagation in a late mound visualized with GCaMP6s (see Supplementary Fig. 1d for corresponding snapshots). Fluorescent images of GCaMP6s were acquired

by the epifluorescence microscope at 20 s intervals. Scale bar, 500 μm.

Movie 7

Live imaging of $[Ca^{2+}]_i$ dynamics through the development of *Dictyostelium* mutant cells lacking *iplA* using GCaMP6s (first half). Data were obtained 3–7.5 hours after starvation. This movie shows the onset of aggregation, early aggregation and the early mounds with streams. Left, DIC image. Right, fluorescence image of GCaMP6s. The images were acquired by an epifluorescence microscope at 20 s intervals. Scale bar, 1 mm.

Movie 8

Disappearance of $[Ca^{2+}]_i$ wave propagation during the multicellular formation (see Fig. 1f for corresponding snapshots). Data were obtained 6.5–9 hours after starvation. Fluorescent images of GCaMP6s were acquired by the epifluorescence microscope at 20 s intervals. Scale bar, 500 μm.

Movie 9

$[Ca^{2+}]_i$ signal in a migrating slug visualized with YC-Nano15 (see Fig. 2a for corresponding snapshots). Fluorescent images of YC-Nano15 were acquired by the confocal microscope at 10 s intervals. Ratio of CFP/YFP signals is shown by psuedocolor. Scale bar, 50 μm.

Movie 10

$[Ca^{2+}]_i$ burst at the tip region of a slug visualized with GCaMP6s (see Fig. 2c for corresponding snapshots). Left, DIC image. Right, fluorescence image of GCaMP6s. The images of GCaMP6s were acquired by the epifluorescence microscope at 20 s intervals. Scale bar, 100 μm.

Movie 11

$[Ca^{2+}]_i$ burst at the posterior region of a slug visualized with GCaMP6s (see Fig. 2e for corresponding snapshots). Left, DIC image. Right, fluorescence image of GCaMPs. The images of GCaMP6s were acquired by the epifluorescence microscope at 20 s intervals. Scale bar, 100 μm.

Movie 12

$[Ca^{2+}]_i$ burst of a slug induced by mechanical stimulation using a plastic rod (see Fig. 3a for corresponding snapshots). The fluorescence images of GCaMP6s were acquired by

the epifluorescence microscope at 5 s intervals. Scale bar, 50 μm.

Movie 13

$[Ca^{2+}]_i$ burst at the tip region of a slug induced by mechanical stimulation using a micropipette (see Fig. 3c for corresponding snapshots). Left, DIC image. Right, fluorescence image of GCaMP6s. The images of GCaMP6s were acquired by the epifluorescence microscope at 15 s intervals. Scale bar, 50 μm.

Movie 14

$[Ca^{2+}]_i$ burst at the posterior region of a slug induced by mechanical stimulation using a micropipette (see Fig. 3d for corresponding snapshots). Left, DIC image. Right, fluorescence image of GCaMP6s. The images of GCaMP6s were acquired by the epifluorescence microscope at 15 s intervals. Scale bar, 100 μm.

Movie 15

$[Ca^{2+}]_i$ burst of a slug induced by mechanical stimulation using a plastic rod with 1mM EGTA containing agar (see Fig. 5a for corresponding snapshots). The fluorescence images of GCaMP6s were acquired by the epifluorescence microscope at 5 second intervals. Scale bar, 100 μm.

Supplementary Tables.

Supplementary Table 1.

Strain name	Characteristics	Background	Source or reference
AX2	wild type	AX2	Lab stock
iplA⁻	*iplA*⁻ (bsR)	AX2	Traynor et al., 2000<br>NBRP-nenkin (ID: S00009)
pzoA⁻	*pzoA*⁻ (bsR)	AX2	This study
GCaMP6s/AX2	(neoR)	AX2	This study
GCaMP6s/*iplA*⁻	*iplA*⁻ (bsR, neoR)	AX2	This study
GCaMP6s/*pzoA*⁻	*pzoA*⁻ (bsR, neoR)	AX2	This study
Flamindo2 /AX2	(neoR)	AX2	Hashimura et al., 2019<br>NBRP-nenkin (ID: S90789)

Supplementary Table 2.

Primer name	Sequences
pzA_KO_LA1	AGCGCGTCTCCAATGCTGCAGCAAGTGTAGCATCAGCGATATTATTAGC
pzA_KO_LA2	AGCGCGTCTCCGTTGTAATATCCAATTGATTAATTGATGCTGA
pzA_KO_RA1	AGCGCGTCTCCCTTCGTGCATTACAAATTTGTTATGGTTATCC
pzA_KO_RA2	AGCGCGTCTCCTCCCCTGCAGAACAGAATTTGAACCAACAATAACCGAA
pzA_up_Fw	GGAAATAAAAAAATGATAGGATATTTCTTTGTG
pzA_down_Rv	TGGTAAAACTGTTTCACAAGTTGCTACTTCC

Supplementary Table 3.

Plasmid name	Characteristics	Backbone	Source or reference
pHK12neo_Dd-GCaMP6s	GCaMP6s	pHK12neo (neoR)	This study
pBIG_YC-Nano15	Yellow Cameleon-Nano15	pBIG (neoR)	Horikawa et al., 2010<br>Addgene (#51962)
pHK12neo_Dd-Flamindo2	Flamindo2	pHK12neo (neoR)	Hashimura et al., 2019<br>NBRP-nenkin (ID: G90480)
pKOSG-IBA-dicty1_pzoA	pzoA disruption (bsR)	pKOSG-IBA-dicty1	This study
